# Rotten to the core: antivirals targeting the HIV-1 capsid core

**DOI:** 10.1186/s12977-021-00583-z

**Published:** 2021-12-22

**Authors:** William M. McFadden, Alexa A. Snyder, Karen A. Kirby, Philip R. Tedbury, Monika Raj, Zhengqiang Wang, Stefan G. Sarafianos

**Affiliations:** 1 Laboratory of Biochemical Pharmacology, Department of Pediatrics, Emory University School of Medicine, Atlanta, GA 30322 USA; 2 Children’s Healthcare of Atlanta, Atlanta, GA 30322 USA; 3 Department of Chemistry, Emory University, Atlanta, GA 30322 USA; 4 Center for Drug Design, College of Pharmacy, University of Minnesota, Minneapolis, MN 55455 USA

## Abstract

The capsid core of HIV-1 is a large macromolecular assembly that surrounds the viral genome and is an essential component of the infectious virus. In addition to its multiple roles throughout the viral life cycle, the capsid interacts with multiple host factors. Owing to its indispensable nature, the HIV-1 capsid has been the target of numerous antiretrovirals, though most capsid-targeting molecules have not had clinical success until recently. Lenacapavir, a long-acting drug that targets the HIV-1 capsid, is currently undergoing phase 2/3 clinical trials, making it the most successful capsid inhibitor to-date. In this review, we detail the role of the HIV-1 capsid protein in the virus life cycle, categorize antiviral compounds based on their targeting of five sites within the HIV-1 capsid, and discuss their molecular interactions and mechanisms of action. The diverse range of inhibition mechanisms provides insight into possible new strategies for designing novel HIV-1 drugs and furthers our understanding of HIV-1 biology.

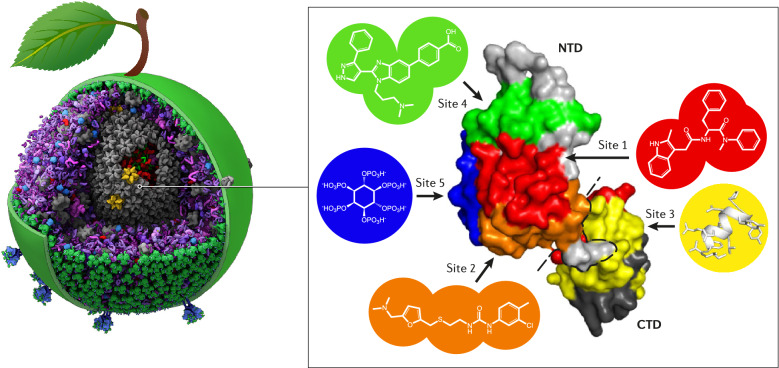

## Introduction

As of 2020, it is estimated that 38 million individuals are living with human immunodeficiency virus type 1 (HIV-1) globally, and of these individuals, over 25 million are receiving antiretroviral therapy (ART) [1]. HIV-1 is a lentivirus that infects CD4^+^ human immune cells, such as T cells and macrophages, and is the causative agent of acquired immune deficiency syndrome (AIDS) [2–4]. The biological structure of the infectious HIV-1 particle, also known as the mature virion, has a viral envelope surrounding a “fullerene cone”-shaped capsid shell that encapsulates two copies of the positive-strand RNA genome along with associated cellular factors and viral proteins [4–9]. The capsid core is composed of capsid proteins (CA), also known as p24, that form pentameric and hexameric subunits, which assemble into the mature viral capsid. In this review we will refer to the capsid protein as “CA” and to the assembled viral conical core as “capsid” or “core”. Further, capsid serves critical roles in many aspects of the HIV-1 replication cycle such as reverse transcription, cytoplasmic transport, nuclear entry, and virion maturation in addition to interacting with over 20 host factors essential for infection [9–14]. These functions are fundamental to HIV-1 biology and therefore there is high interest in developing drugs that perturb capsid functions [15, 16]. Thus, this review is focused on the structure of CA and the compounds that specifically target it.

HIV-1 infection begins upon binding of the virion to a CD4 receptor and a CCR5 or CXCR4 coreceptor on the target cell surface. Receptor binding triggers a fusion event of the viral envelope with the host cell membrane, releasing the viral capsid and its contents into the cytoplasm [17, 18]. The precise timing, rate, and location at which the CA proteins are shed, a process at times referred to as “uncoating”, is still under investigation. Uncoating kinetics appear to be highly regulated, as mutations that either stabilize or destabilize CA–CA association have a negative impact on viral infectivity [19]. Regardless, it is known that CA serves multiple roles during and following reverse transcription, including trafficking to and through the nuclear pore complex (NPC), as well as integration of viral DNA into the host cell genome [12, 20–24]. Reverse transcription, the process of converting the HIV-1 RNA genome into double-stranded cDNA, is catalyzed by the enzyme reverse transcriptase (RT) located within the capsid, which prevents exposure of the viral nucleic acid to host proteins [12, 25]. For trafficking of viral particles to the NPC, CA interacts with the microtubule-associated proteins MAP1A and MAP1S, along with FEZ1, a kinesin-1 adaptor protein, and others [26–30]. At the NPC, CA interacts directly with nuclear pore proteins, including NUP358 and NUP153, both of which are essential for proper nuclear import [31–34]. Once the viral cDNA is inside the nucleus, integration can occur [35–38]. Integration is the incorporation of the viral cDNA, the product of reverse transcription, into the host cell’s genome forming the provirus, a process that is catalyzed by the HIV-1 enzyme integrase (IN) [39–41].

Provirus formation marks the end of the early phase of the HIV-1 life cycle [42]. The provirus is used as a template for transcription of nascent viral RNAs that may be translated into viral proteins or packaged as a viral genome. In some instances, the provirus enters a latent state where it remains transcriptionally inactive, enabling evasion of the immune response until reactivation of the host cell triggers expression of viral RNA [43, 44].

While CA does not have known catalytic activity, it can impact multiple viral enzymatic activities, including that of RT and IN, by mechanisms that are currently under investigation [11–13, 45–48]. Productive infection is further influenced by interactions of CA with host factors, including Cyclophilin A (CypA) and cleavage and polyadenylation specificity factor subunit 6 (CPSF6). Although these proteins bind CA at different sites, both CypA and CPSF6 have been shown to increase viral fitness and impact the location of integration events [34, 49–56]. This example is a limited view into the many complex roles of cellular factors for infection (recently reviewed in [7, 9, 57]).

The late phase of the HIV-1 life cycle begins with transcription of the provirus, followed by export of the synthesized RNA to the cytoplasm and translation of the Gag and GagPol polyproteins [42, 44, 58]. The Gag polyprotein precursor, also known as Pr55^Gag^, contains four major domains: Matrix (MA), CA, Nucleocapsid (NC), and p6, in addition to two small spacer peptides: SP1 and SP2 [59, 60]. In total, the molecular weight of Gag is ~ 55 kDa [61]. GagPol is a larger polyprotein precursor that is translated as a result of a programmed + 1 ribosomal frameshift in ~ 5% of cases compared to the relatively shorter Gag polyprotein [61, 62]. GagPol contains the aforementioned domains of Gag, except for a transframe domain that replaces the p6 domain, known as p6* or p6pol, following the frame shift located C-terminal region of NC [62]. Additionally, GagPol includes the protease (PR), RT, and IN domains at the C-terminal end, giving rise to a 160 kDa protein [60, 62].

Following translation, Gag proteins are trafficked to the inner leaflet of the plasma membrane [63, 64]. This localization is guided by the N-terminal MA domain that forms specific interactions with phosphatidyl-4,5-bisphosphate (PI(4,5)P_2_) and utilizes a myristic acid moiety as a membrane anchor [65–70]. The Gag protein interacts with the viral RNA at the plasma membrane; this is thought to nucleate Gag multimerization and particle assembly [61, 71]. Further, CA domain interactions serve as the driving force for this multimerization process, thus assembling the immature Gag lattice (reviewed in [61]). Due to the structural flexibility, the size, and the curved structure of the Gag-lattice, obtaining high-resolution structures of this protein assembly has posed a challenge. A low resolution structure of this lattice in virus-like particles was solved using cryogenic-electron tomography (cryo-ET) revealing that the lattice was formed from hexameric structures [72, 73]. An essential structural element of the Gag lattice is the CA-SP1 junction. The C-terminal residues of CA extended by SP1 form a dynamic 6-helix bundle that is necessary for the formation of the Gag lattice [74–77]. These interactions are further stabilized by the highly abundant metabolite inositol hexaphosphate (IP6) [78–80]. This site is also proposed to be a binding site for HIV-1 maturation inhibitors [61]. Several additional steps follow lattice assembly, including RNA packaging and envelope protein (Env) incorporation [81, 82], all leading to immature viral particles that bud from the plasma membrane, thus releasing the virion to the extracellular space [83].

The maturation process begins concomitantly with, or shortly following, budding [84–86]. Maturation is triggered by the PR cleavage of the Gag polyprotein. Gag processing is a multi-step process, the first of which involvescleavage between the SP1 and NC domains, followed by a series of cleavage events that further fragment Gag and Gag-Pol into individual proteins [61, 87]. Following polyprotein processing, HIV-1 maturation is characterized by large-scale structural rearrangements that lead to formation of the capsid, whereby CA hexamers and pentamers encapsidate two copies of the viral genomic RNA and other proteins, including RT, PR, and IN. The process of capsid maturation is incompletely understood, although there are several proposed mechanisms. The first and more widely supported mechanism hypothesizes that mature capsid reassembles from freed CA monomers present in the interior of the HIV-1 virion [88–90]. Another proposed mechanism posits that the mature capsid lattice forms without the freeing of CA monomers in the virus particle, called the displacive transition model [86, 91]. An alternative mechanism for capsid assembly combines elements from both proposed mechanisms [92].

After reassembly, the mature “fullerene cone”-shaped capsid shell comprises roughly 1500 CA proteins arranged in approximately 250 hexamers and precisely 12 pentamers [76, 93–96]. The pentamers are located on highly curved areas of the capsid core; specifically, seven pentamers are located at the wide end and five pentamers are located at the narrow end of the mature capsid. It is unknown whether it is the location of the pentamers that dictate the shape of the capsid or if it is the curvature of the capsid that determines the placement of the pentamers [61, 95, 97].

**Fig. 1:**
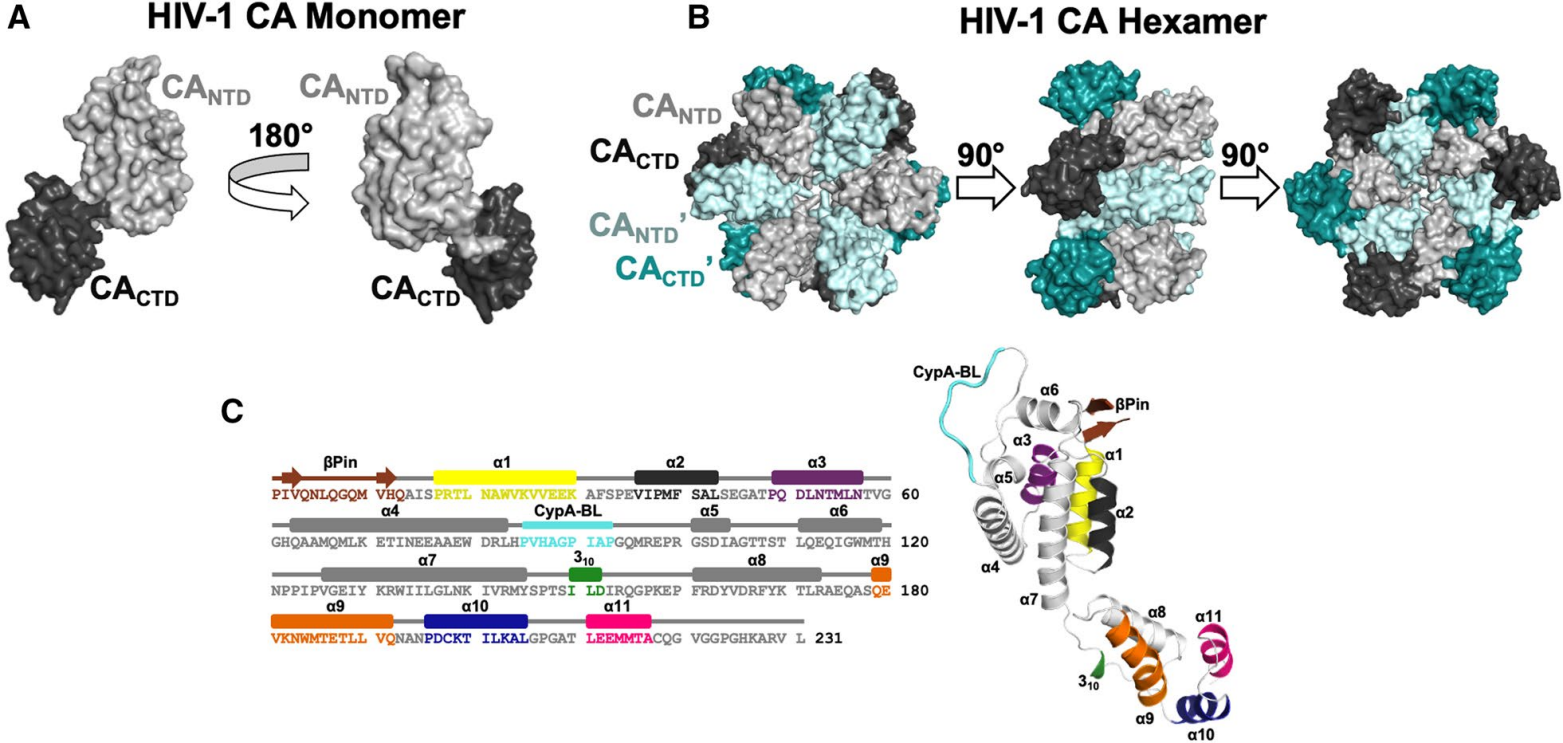
Structure of mature HIV-1 CA. **A** The HIV-1 CA monomer comprises CA_NTD_ (light gray) and CA_CTD_ (dark gray) domains (PDB ID: 4XFX). The view is rotated by 180°. **B** The same domains are shown in the context of the HIV-1 CA hexamer. Colors are as described in **A**, with neighboring CA_NTD_ʹ and CA_CTD_ʹ domains shown as light and dark cyan, respectively. The views are rotated by 90°. **C** Schematic outlining the sequence and secondary structural elements of HIV-1 CA (based on PDB ID: 4XFX) (Part **C** of this figure is based on Fig. 1 from Gres et al. [105]. Reprinted with permission from AAAS)

Individual CA monomers contain helical N-terminal domains (CA_NTD_) and C-terminal domains (CA_CTD_) connected through a flexible linker [93, 94, 98–106]. Specifically, CA_NTD_ contains seven α-helices (α1-7) and CA_CTD_ contains one short 3_10_-helix and four α-helices (α8-11) (Fig. 1). Key structural elements of CA include an N-terminal β-hairpin that forms after proteolytic cleavage of the N-terminus, a proline-rich CypA-binding loop (CypA-BL) that interacts with human proline isomerase CypA, an interdomain linker, and a sequence known as the major homology region (MHR) which is conserved among orthoretroviruses [61, 97, 107, 108]. CA domains form interactions at the two-, three-, and sixfold symmetry regions of CA hexamers. These interactions are necessary for proper construction of the complete conical capsid. The central ring of the capsid hexamers is composed of six CA_NTD_ domains facing each other and held together by intrahexameric CA_NTD_–CA_NTD_ and CA_NTD_–CA_CTD_ interactions [98, 105]. It has also been reported that IP6 can interact with electropositive residues of this pore to stabilize the CA hexamers, thereby stabilizing the capsid core [80]. Along with stabilization, IP6 is thought to facilitate transport of deoxynucleotide triphosphates (dNTPs) into the capsid. IP6’s cellular functionality and interactions with capsid are topics of current research [80, 109–112]. In contrast to the inner hexamer ring, the outer ring of each hexamer is made up of six CA_CTD_s. The exteriors of each CA hexamer can then make interactions with neighboring CA hexamers. These outer C-terminal domains participate in interhexamer interactions that involve CA residues at the two- and threefold symmetry axes [93, 98, 102–104, 113]. The capsid remains in this conformation until the next infection is established.

Understanding the structural interactions at the interfaces of these capsid building blocks is crucial for learning about the dynamics that regulate capsid assembly and disassembly. Towards that end, structural biology techniques such as X-ray crystallography, cryogenic electron microscopy (cryo-EM), 3D nuclear magnetic resonance (NMR), and cryo-ET have been used. CA hexamers and pentamers, in cross-linked form, were first studied using X-ray crystallography. These early studies laid the foundation for the solution of many CA structures and revealed essential intrahexameric interactions [98, 102]. The X-ray structure of native CA in hexameric form revealed structural information of interhexameric interactions at the three- and twofold symmetry interfaces [105]. These CA–CA interactions are critical for the structural integrity and stability of the core [19, 91, 93, 94, 98, 102, 114–123]. Important low resolution information provided by cryo-EM in combination with NMR led to early useful molecular models of HIV-1 capsid [94]. Using new cryo-EM methodologies (ArbitrEM), a high-resolution structure of CA hexamers in tubular assemblies was solved [124]. Of note, cryo-EM may better recapitulate the curved nature of the core [97, 124]. Moreover, there are additional recent structures of CA tubes studied by cryo-ET [94, 97], cryo-EM or solid state NMR [94, 124–126]. Collectively, decades of structural work laid the foundation for understanding capsid structure and interactions that led to the discovery of numerous CA-targeting antivirals.

The interactions between CA proteins are the key determinants for the stability of the mature capsid, which is essential for the precise timing of the assembly and uncoating steps in the HIV-1 life cycle. Thus, creating small molecules that either stabilize or destabilize the capsid core is a promising strategy for the discovery of novel antiviral treatments.

## Targeting HIV-1 by antivirals that bind to distinct sites of CA

Currently, there are 49 FDA-approved HIV medicines [127] that fall into seven categories: protease inhibitors (PIs), integrase strand transfer inhibitors (INSTIs), non-nucleoside reverse transcriptase inhibitors (NNRTIs), nucleoside (or nucleotide) reverse transcriptase inhibitors (NRTIs), fusion inhibitors, chemokine receptor antagonists, attachment inhibitors, and post-attachment inhibitors [128–132]. Although these drugs provide life-saving treatments for millions of individuals, there is no cure or vaccine presently available for HIV. Further, ART has several limitations including drug resistance, virologic failure, viral transmission, toxicity, and limited treatment options for treatment-experienced individuals [133, 134]. Therefore, it is essential to produce novel HIV drugs and to continue research on new therapeutic targets.

**Fig. 2:**
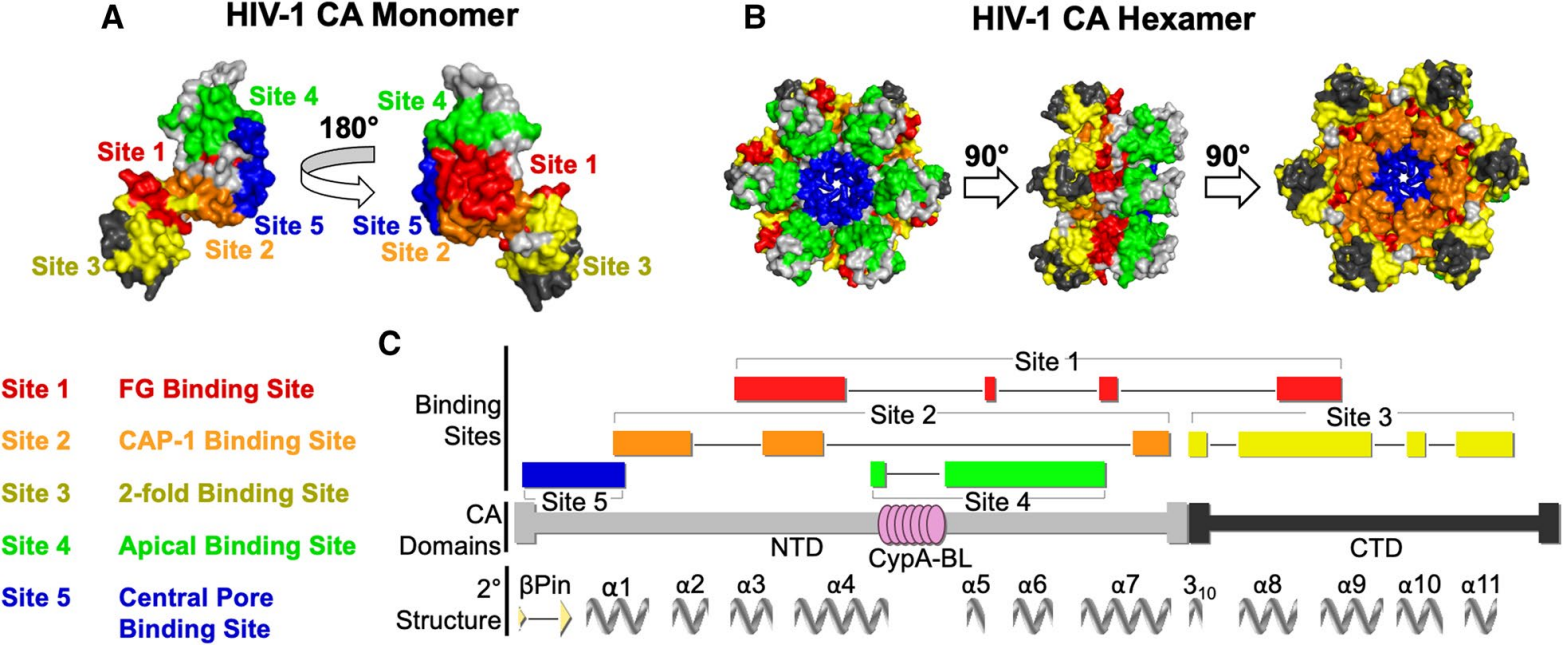
Binding sites of CA-targeting antivirals in mature HIV-1 CA monomers and hexamers. **A** The five CA-targeting antiviral binding sites are shown mapped onto the HIV-1 CA monomer (PDB ID: 4XFX). CA_NTD_s are shown in light gray, CA_CTD_s are shown in dark gray. Binding site 1 is shown in red, binding site 2 in orange, binding site 3 in yellow, binding site 4 in green, and binding site 5 in blue. The view is rotated by 180°. **B** The same domains and five CA-targeting antiviral binding sites are shown mapped onto the HIV-1 CA hexamer. Colors are as described in **A**. The views are rotated by 90°. **C** Schematic outlining the domains, binding sites, and secondary structure of HIV-1 CA. Compound binding sites are colored as described in **A**, and the Cyclophilin A binding loop (CypA-BL) is shown as pink circles. The CA_NTD_ and CA_CTD_ are shown as light and dark gray bars, respectively. The β-hairpin (βPin) and α-helices (α) shown as gray arrows and twists, respectively. Schematic generated by Protean 3D™ (V17.2.1) from DNASTAR, Inc

As such, the HIV-1 capsid has emerged as the next target of novel antiretrovirals due to its essential roles in both the early and late stages of the viral replication cycle [135]. Potential capsid effectors may interfere with CA–CA interactions within interhexameric or intrahexameric assemblies [136]. Such interference may modify the stability and morphology of the mature capsid, thus disrupting the processes of assembly and/or disassembly and resulting in the suppression of viral infectivity. Below, we review compounds that bind at five distinct sites of the CA protein (Fig. 2).

## Site 1: FG binding site

**Fig. 3:**
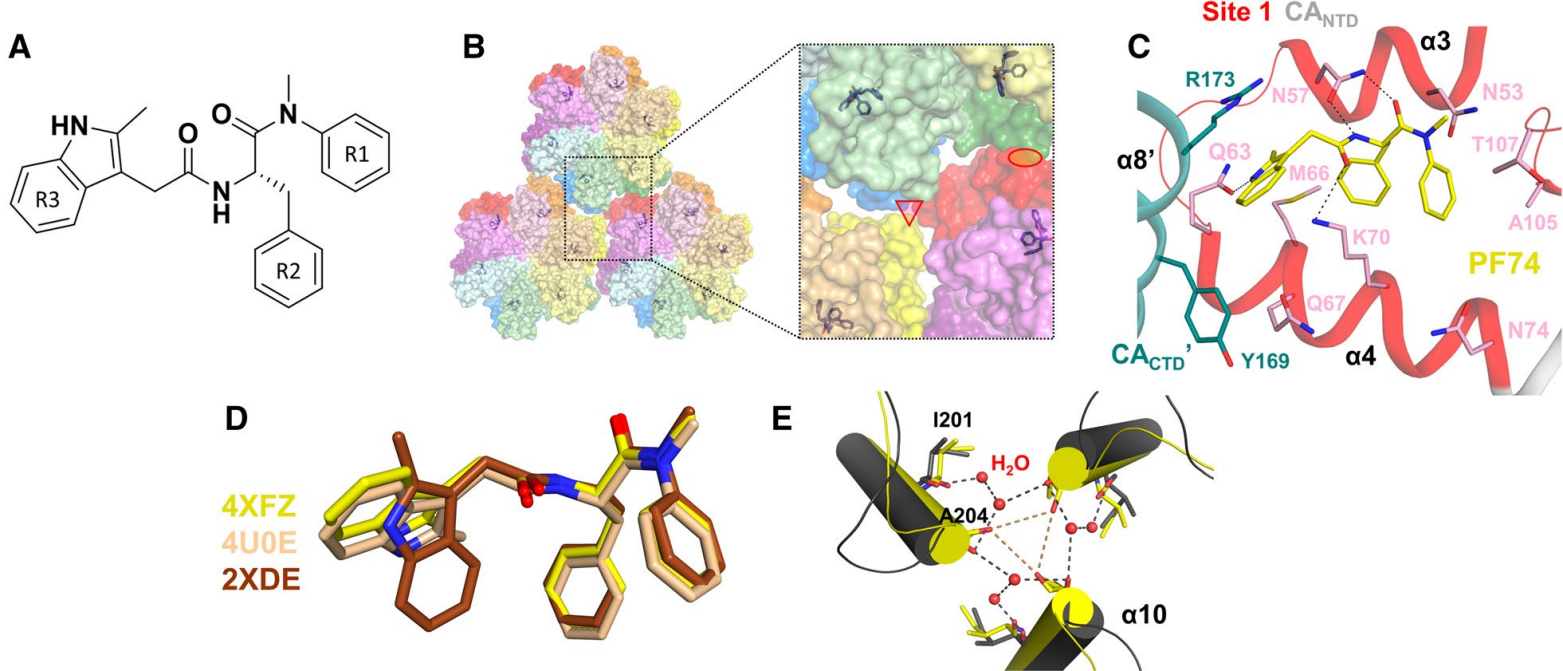
Binding interactions of PF74 at binding site 1 of HIV-1 CA. **A** Chemical structure of PF74. **B** PF74 binding at the CA_NTD_–CA_CTD_ interfaces of adjacent subunits within a CA hexamer (PDB ID: 4XFZ). The larger box shows a close-up view of the PF74 binding site in relation to the threefold (triangle) and twofold (oval) interfaces. CA_NTD_s and the corresponding CA_CTD_s are colored by the same colors (light and dark, respectively). **C** CA_NTD_ and a CA_CTD_ of a neighboring subunit (CA_CTD_ʹ) participate in PF74 binding (gray and dark cyan ribbons; site 1 residues are shown in red cartoon and pink sticks). PF74 is shown as yellow sticks. **D** PF74 binding geometries in CA-PF74 (PDB ID: 4XFZ; PF74 in yellow), CA_XL_-PF74 (PDB ID: 4U0E; PF74 in beige), and CA_NTD_-PF74 (PDB ID: 2XDE; PF74 in brown). **E** Threefold interface of CA-PF74 superposed onto CA (aligned based on residues 1–219). Helices α10 of CA-PF74 and CA are in yellow and dark gray. Water molecules are shown as red spheres in CA; no water molecules were present in CA-PF74 (Parts of this figure are based on Fig. 4 from Gres et al. [105]. Reprinted with permission from AAAS)

The FG binding pocket is a primarily hydrophobic pocket that comprises structural elements of neighboring capsid monomers within a CA hexamer (Figs. 2, 3). Binding site 1 is formed by residues from the α3 and α4 α-helices of a CA_NTD_ subunit and by residues from the α8ʹ and α9ʹ α-helices of the CA_CTD_ of the adjacent subunit (Fig. 1) [105, 137–139]. This site has been the target of several small molecule antivirals. The compounds that bind CA at this pocket can compete with host factors that also bind at the same site, such as NUP153, CPSF6, and Sec24C, through interactions of phenylalanine–glycine (FG)-motifs present in these cellular proteins, hence the name of the pocket. Perturbation of NUP153, CPSF6, and Sec24C interactions with CA adversely affect nuclear import and viral infectivity [31, 32, 140, 141]. This pocket is also the binding site of small molecules that are discussed below: PF74 and analogs, BI-1 and BI-2, GS-CA1 and GS-6207, and others (Tables 1, 2).

**Table 1:**
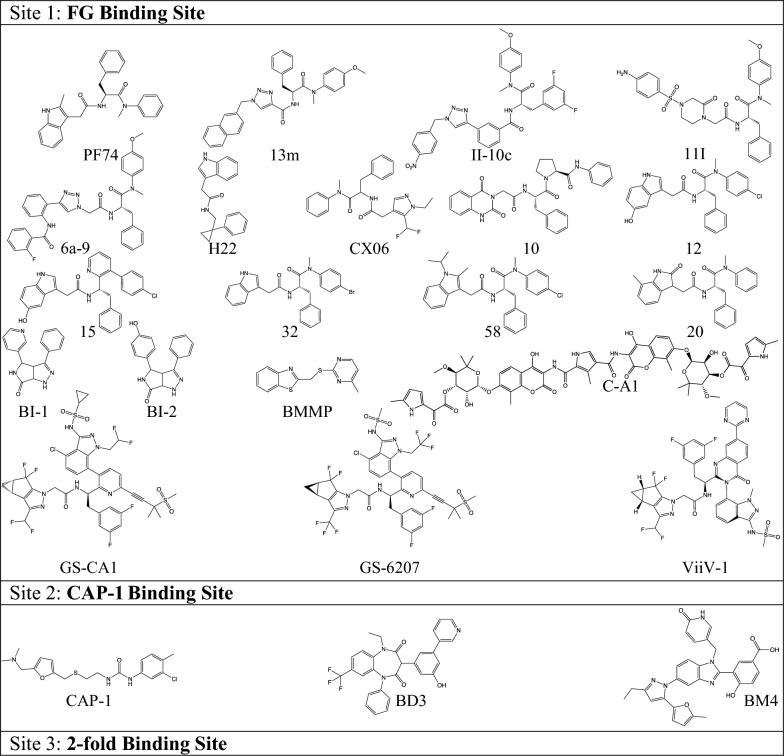

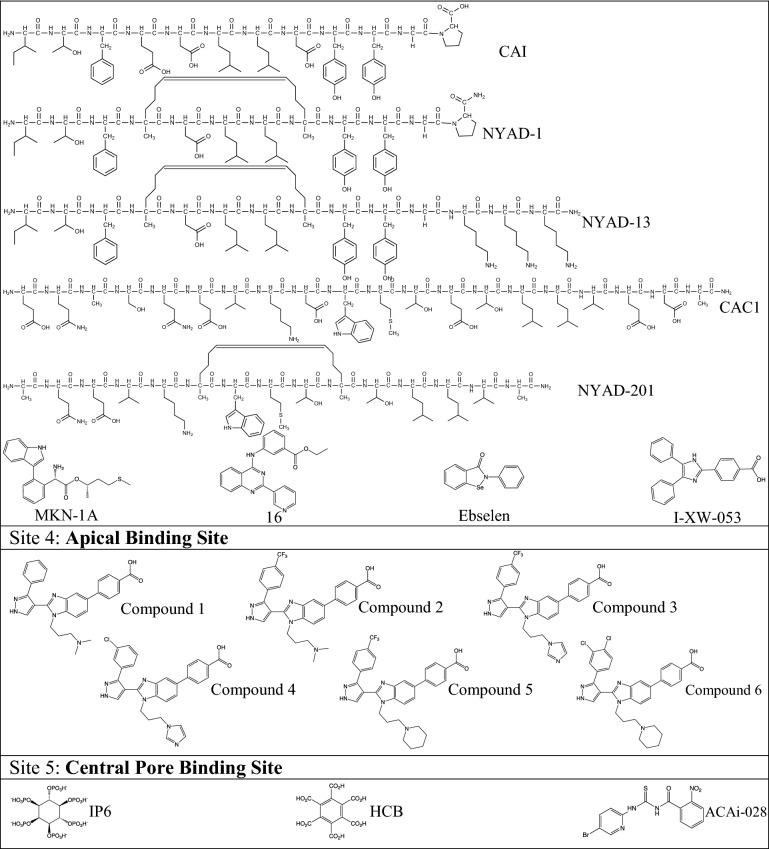
Chemical structures of each CA-targeting antiviral discussed

**Table 2:** Compounds targeting binding site 1: FG binding site

Compound and citation(s)	Key interactions	Effects on stability and/or assembly	HIV-1 stage	IC_50_^a^/EC_50_	CC_50_
PF74 [137]	N53, L56, N57, V59, Q63, M66, Q67, L69, K70, I73, A105, T107, Y130	Stabilize, increase multimerization	Early/late	0.57 µM	69 µM
13m [149]	N/A	N/A	N/A	4.33 µM	57.7 µM
II-10C [153]	C14, I15, P17, L20, N21, Q50, T54, N57, T58, G60, K70^b^	N/A	N/A	2.13 µM	35.5 µM
11I [154]	Q50, N53, T54, L56, N57, K70, N74, G106^b^	Decrease multimerization	Early/late	0.09 µM	34.5 µM
6a-9 [155]	N53, L56, N57, M66, K70, I73, N74, A105, T107^b^	No effect on multimerization	Early/late	3.13 µM	16.5 µM
H22 [156]	L56, N57, Q63, M66, L69, K70, I73, Y130, Y169, L172, R173, K182^b^	Increase multimerization	N/A	18.1 µM^a^	526 µM
CX06 [150]	N/A	Destabilize, decrease multimerization	Early/late	5.9 µM^a^	> 100 µM
10 [157]	P34, N53, L56, N57, L59, Q63, M66, K70, Y130, R173, Q179, K182^b^	Stabilize	N/A	1.6 µM	> 100 µM
12 [158]	N53, N57, Q63, K70, N74, G106, Y130, R173, Q179, K182^b^	Stabilize	N/A	0.032 µM	> 100 µM
15 [159]	N57, Q63, K70, T107, K182^b^	Stabilize	N/A	0.31 µM	44 µM
20 [151]	N53, N57, Q63, K70, G106, T107, Y130, R173, Q179, K182^b^	Stabilize	N/A	0.88 µM	> 50 µM
32 [152]	N53, N57, Q63, Q67, KN74, K70, Y130, Y169, R173, Q179, K182^b^	Stabilize	N/A	0.14 µM	45 µM
58 [152]		Stabilize	N/A	0.15 µM	14 µM
BI-1 [160]	N53, L56, N57, M66, L69, K70, I73, N74, A105, G106, T107, Y130	Stabilize, increase multimerization	Early/late	8.2 µM	> 92 µM
BI-2 [160]		Stabilize, increase multimerization	Early/late	1.8 µM	> 43 µM
BMMP [173]	N/A	Decrease multimerization	Late	25 µM^a^	N/A
C-A1 [174, 175]	Q50, N53, N57, N63, Q67, N70, N74, A105, T107, N112, E128, R173, Q179, K182^b^	N/A	Early	0.98 µM^a^	8.0 µM
GS-CA1 [162]	L57, N57, M66, Q67, K70, N74, T107^b^	Increase multimerization	Early/late	240 pM	N/A
GS-6207 [135]	N57, K70, N74, N183	Increase multimerization	Early/late	95 pM	> 50 µM
ViiV-1 [170–172]	N/A	N/A	Early	25 pM	N/A

Compound stabilizing effects on HIV-1 CA were determined by thermal shift assays and compounds effects on capsid assembly were determined by in vitro capsid assembly assays

*N/A* not available

^a^Refers to IC_50_

^b^Refers to key interactions found through non-structural studies such as molecular docking

### PF74

PF-3450074 (PF74) (Fig. 3A) is a peptidomimetic capsid-targeting antiviral developed by Pfizer, USA (Tables 1, 2) [137]. This compound was shown to inhibit HIV-1 replication [52, 142, 143] with a submicromolar EC_50_ [137]. PF74 has a bimodal inhibition mechanism of action, whereby at lower concentrations (~ 2 µM or less), it interferes with CA-host factor interactions, whereas at concentrations greater than 10 µM, it triggers premature uncoating and blocks reverse transcription [144]. Structural studies of PF74 with CA_NTD_ [137], cross-linked CA hexamers [139], or native CA [105] have shown that PF74 binds at binding site 1 (Fig. 3B, C). Specifically, the R1 group of PF74 makes hydrophobic interactions with I73, A105, T107, T130 and a stacking interaction with N53. The benzyl group of R2 interacts with hydrophobic residues M66, L69, V59, I73, and L56. The R3 indole group interacts with the residues M66, Q67, K70, and Q63; the indole NH also forms a hydrogen bond with Q67 via a water molecule. Also, the amide bond of PF74 forms a hydrogen bond with N57 [137]. Intriguingly, the binding mode of PF74 to CA_NTD_ monomer is somehow different than its binding mode to the interface of adjacent CA monomers (Fig. 3D). Antiviral binding at this site is shared and competed for with the binding of host factors, including NUP153, Sec24C, and CPSF6 [32, 138, 140, 145]. PF74 has been known to affect both the stability of the capsid and the rate of CA assembly in vitro [138, 146]. Of note, the antiviral activity of PF74 is enhanced in the presence of CypA [144]. Though the mechanism has not been fully elucidated, there is antagonistic relationship between PF74 and cyclosporin, the molecules that bind CypA and sequester it from potential CA interactions, which may perturb other host factor interactions that PF74 mimics [34, 142, 143]. The structure of native CA in complex with PF74 has provided insight on the molecular interactions that may lead to stability changes of the capsid core; PF74 binding is accompanied with changes at the hydration layer and interactions between CA hexamers at the threefold interface of the CA lattice [105] (Fig. 3E).

**Fig. 4:**
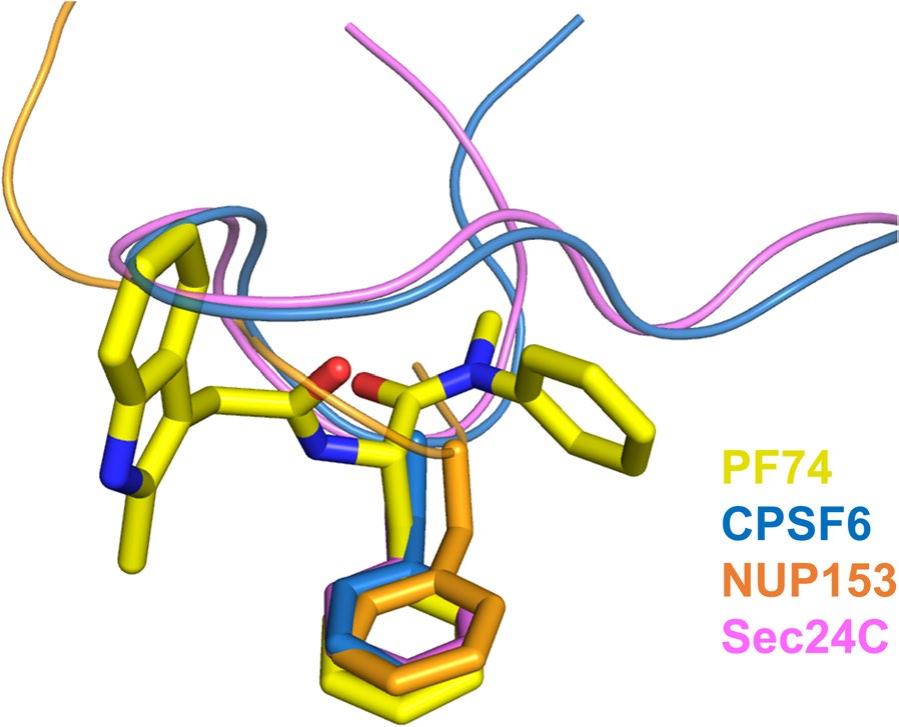
Host factor FG motifs share a common binding conformation with the PF74 benzyl group. Crystal structures of HIV-1 CA hexamers in complex with CPSF6 (PDB ID: 6AY9; blue cartoon and sticks), NUP153 (PDB ID: 6AYA; orange cartoon and sticks), and Sec24C (PDB ID: 6PU1; magenta cartoon and sticks) peptides were aligned to a crystal structure of HIV-1 CA hexamer in complex with PF74 (PDB ID: 4XFZ; yellow sticks). The HIV-1 CA protein is omitted for clarity. The benzyl group of PF74 binds in a similar manner to the benzyl group of the phenylalanine in the FG motif of each host factor peptide

The structure of PF74 comprises three aromatic moieties: R1 is based on a phenyl ring, R2 is a benzyl group, and R3 is a substituted indole ring (Fig. 3A). Comparison of the crystal structures of CA in complex with short peptides derived from CPSF6, NUP153, and Sec24C and of CA in complex with PF74 show that the peptides of the host factors interact with binding site 1 using their FG motifs, although the bound peptides had additional interactions (Fig. 4). The R2 benzyl group of PF74 serves as the surrogate for the phenylalanine group of the FG motif (Fig. 4) (reviewed in [7]).

Several point mutations, such as S41A, Q67H, K70R, and T107N [143], have been identified in CA that decrease PF74 efficacy, in addition to decreasing the requirement for NUP153 interactions and generally decreasing HIV-1 infectivity [143, 147, 148]. Furthermore, PF74 has a very short half-life that predicts excessive first pass liver metabolism and poor oral bioavailability [149–151], making it a less-than ideal therapeutic compound. However, there have been recent advances in PF74-derived compounds. Chemical profiling has identified important characteristics of PF74 for developing further analogs, which include, for example, benzene rings with *para*-halogen substitutions and alkylation at the N-1 position of the indole ring [152]. Several reports have identified PF74-derived compounds that bind the same pocket, are more potent inhibitors of HIV-1, and have longer half-lives than PF74. The best performing compounds include 13m [149], II-10c [153], 11l [154], 6a-9 [155], H22 [156], CX06 [150], 10 [157], 12 [158], 15 [159], 20 [151], 32 [152], and 58 [152] (Tables 1, 2).

### BI-1 and BI-2

**Fig. 5:**
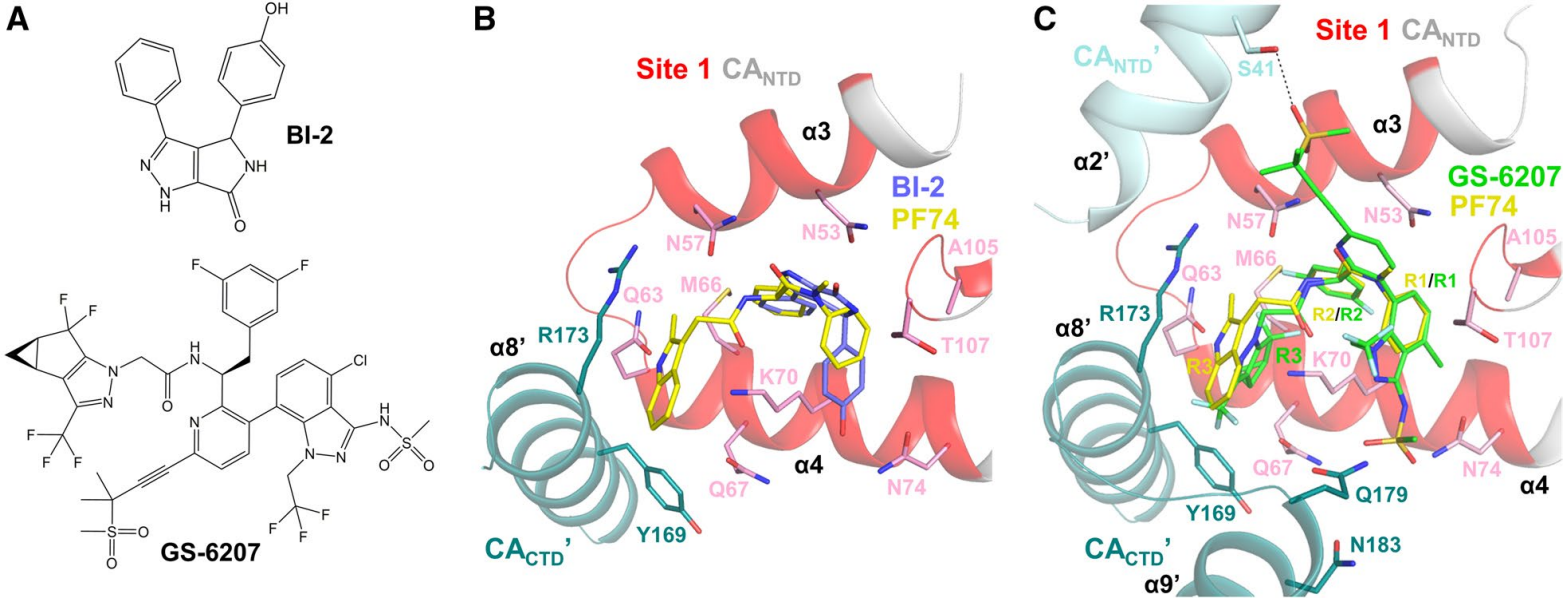
Different inter-subunit interactions of various CA-targeting compounds at CA binding site 1. **A** Chemical structures of BI-2 and GS-6207 (PF74 is shown in Fig. 3A). **B** BI-2 only interacts with the N-terminal domain of HIV-1 CA. The crystal structure of HIV-1 CA hexamer in complex with BI-2 (PDB ID: 4U0F; purple sticks) was superposed to a crystal structure of HIV-1 CA hexamer in complex with PF74 (PDB ID: 4XFZ; yellow sticks). The CA_NTD_ of one CA monomer is shown in light gray cartoon with binding site 1 shown in red, the CA_CTD_ of a neighboring monomer (CA_CTD_ʹ) is shown in dark green. Residues interacting with PF74 and/or BI-2 are shown as sticks. **C** GS-6207 forms extended interactions with the CA_NTD_ and CA_CTD_ of a neighboring CA monomer (CA_NTD_ʹ show in light cyan cartoon and CA_CTD_ʹ shown in dark green). The crystal structure of HIV-1 CA hexamer in complex with GS-6207 (PDB ID: 6V2F; green sticks) was superposed to a crystal structure of HIV-1 CA hexamer in complex with PF74 (PDB ID: 4XFZ; yellow sticks). The CA_NTD_ of one CA monomer is shown in light gray cartoon with binding site 1 shown in red. The novel interaction with CA_NTD_ʹ is shown as a black dotted line. The R1 and R2 groups of PF74 (see Fig. 3A) superpose well to GS-6207, while the R3 group adopts a different conformation

4,5-Dihydro-1H-pyrrolo[3,4-c]pyrazol-6-one (pyrrolopyrazolone) antivirals were identified from a screen of compounds that impact the post-entry events of the HIV-1 life cycle [160]. From this screen, two lead compounds were reported: BI-2 (Fig. 5A) and BI-1 (Tables 1, 2). These compounds were found to stabilize capsid in vitro and impair nuclear entry. BI-1 and BI-2 were found to have EC_50_ values of 7.5–8.2 µM and 1.4–1.8 µM, respectively. Crystal structures and ^1^H–^15^N heteronuclear single quantum coherence (HSQC)-NMR revealed that BI-1 binds to the CA_NTD_ and interacts with the α4, α5, and α7 helices [160]. Although they bind at the same site as PF74, the BI compounds differ from PF74 as they only interact with the CA_NTD_ and not the CA_CTD_ (Fig. 5B). BI-1 and BI-2 are active only during the early phase of the HIV-1 life cycle [160], whereas PF74 is active in both early and late phases [137]. Similar to PF74, BI-2 was shown to decrease CPSF6 binding to CA [137, 161].

### GS-CA1 and GS-6207

Developed by Gilead Sciences, both GS-CA1 and GS-6207 (lenacapavir) are complex molecules that contain a polyphenyl core and a linker region scaffold similar to PF74 (Fig. 5A) [162]. The three aromatic group regions of PF74 (R1, R2, and R3) are superposable with related structural elements of GS-CA1 or GS-6207 (Figs. 2A, 5A, C and Tables 1, 2). GS-CA1 and GS-6207 target the CA_NTD_ where FG-containing host factors interface with the CA hexamers and inhibits HIV-1 replication at post-entry steps, as well as production and maturation in virus-producer cells [141, 162]. GS-CA1, compared to PF74, is remarkably more potent (EC_50_ of 240 pM compared to ~ 600 nM), far more metabolically stable, less soluble, and has an outstanding EC_50_/CC_50_ selectivity index. Its attributes made it more appropriate for testing as a potential long-acting antiviral. Hence, when administered subcutaneously to a mouse model, one dose of GS-CA1 was able to maintain compound levels above its EC_50_ in the blood plasma for 56 days, outperforming the long-lasting NNRTI, rilpivirine, in potency and selectivity. During this study, all mice sustained viral suppression except for one who experienced virological failure at week 12 due to the mutations Q67H and K70R [162]. Mutations in CA residues L56I, N57S, M66I, Q67H, K70N, N74D, Al05T, and T107N have also been reported during in vitro resistance selection experiments with GS-CA1 exposure; the magnitudes of resistance vary from 2.3- to > 100-fold resistance [142, 144, 163]. Several of these CA mutations cause PF74 resistance and are located within the PF74 binding pocket [143, 148]. Some GS-CA1 resistance mutations significantly affect viral fitness, making their barrier to resistance higher. Overall, the studies with GS-CA1 showed excellent potential for its use as a long-acting antiviral therapeutic and led to the development of the more potent and metabolically stable GS-6207.

GS-6207 is the first capsid-targeting antiviral to make it into phase III clinical trials [135, 164]. Having a similar structural and mechanistic profile to GS-CA1, GS-6207 (Tables 1, 2, Fig. 5A) also has a remarkable potency, is very metabolically stable, and has an outstanding selectivity index. A crystal structure of GS-6207 bound to a cross-linked CA hexamer shows extensive hydrophobic and electrostatic interactions, seven hydrogen bonds, and two cation-π interactions between GS-6207 and residues of binding site 1 [135] (Fig. 5C). GS-6207 inhibits both early and late stages of the HIV-1 replication cycle, with exceptional EC_50_ values (~ 30 pM in target cells and ~ 440 pM in producer cells). The higher potency in early stages of the viral replication may be due to direct competition with host-cell nuclear import factors, such as NUP153 and CPSF6. In phase I clinical trials, a single ascending dose of GS-6207 was proven to be safe, well-tolerated, and demonstrated slow, sustained drug release, suggesting that a less frequent dosing regimen would be needed [135, 164, 165]. Phase 1b trials produced similar results in safety and effectivity. After 9 days of treatment, a single 450 mg dose of GS-6207 reduced the viral load in plasma by up to 2.2 log_10_. Notably, during this trial, treatment with GS-6207 led to the emergence of the Q67H resistance mutation [135]. Other mutations have appeared in in vitro resistance selection experiments against GS-6207, including L56I (239-fold resistance), M66I (> 3200 to > 85,000-fold resistance), Q67H (six- to tenfold resistance), K70N (24-fold resistance), N74D/S (22-fold resistance), Q67H/T107N (62-fold resistance), and Q67H/N74D (1099-fold resistance) double mutant. Both single and double resistance mutations have arisen from these studies, with M66I posing the greatest resistance to GS-6207 [125, 135], although the fitness of this virus is significantly suppressed. Recently, molecular dynamics simulations suggested that the mechanism of M66I resistance to GS-6207 is primarily a result of the high free energy cost required to reorganize the I66 side chain in order to minimize CA-GS-6207 clashes rather than reduced protein–ligand interactions [166]. Phase II clinical trials (NCT04143594) studied the safety and efficacy of orally and subcutaneously administered GS-6207 in combination with antiviral agents, tenofovir alafenamide (TAF) and emtricitabine. Currently, optimized 6-month oral administration regimens of GS-6207 are underway to evaluate the antiviral activity of this molecule on HIV-1 treatment-experienced patients with failing drug regimens due to multiple drug resistance (MDR) (NCT04150068) [167, 168]. The ongoing clinical trials with GS-6207 provide important validation that capsid is an effective antiviral drug target. Of note, a series of compounds structurally related to GS-6207 were also introduced through scaffold hopping, a computer-aided search for active compounds that contain different core structures [169], and were shown to also have picomolar potency [170–172]. Among them, compound ViiV-1 had an impressive EC_50_ of 25 pM (Tables 1, 2).

### Other antivirals

In addition to the above compounds where binding information is provided by experimental structural approaches, two other compounds have been proposed to suppress HIV-1 replication by interacting at binding site 1 of CA. Specifically, 2-(benzothiazol-2-ylmethylthio)-4-methylpyrimidine (BMMP) (Tables 1, 2) was found to perturb Gag–Gag interactions through a yeast two-hybrid screen of membrane-associated Gag polyproteins [173]. Although the structure of CA-BMMP is not known, it was deduced that BMMP-mediated inhibition occurs through the CA domain of Gag and that the mechanism of action for BMMP is similar to PF74; both compounds impact events following cellular entry but prior to (or in conjunction with) nuclear entry [16, 137, 142, 173]. Additionally, coumermycin-A1 (C-A1) (Tables 1, 2) was shown by Fassati and colleagues to block HIV-1 by interfering with post-nuclear entry steps of the viral life cycle [174, 175]. In this work, it was shown that HIV-1 with A105S and N74D CA mutations conferred resistance to the integration block mediated by C-A1.

## Site 2: CAP-1 binding site

Binding site 2 is located at the CA_NTD_, proximal to binding site 1 (Fig. 2). This site has been targeted by the small molecule CAP-1 as well as various benzodiazepine and benzimidazole compounds. These antivirals bind at the apices of CA α-helices α1, α2, α3, α4, and α7 (Fig. 1). Inhibitor binding either displaces several residues within this site or it protrudes out of the pocket, thereby interfering with capsid assembly and preventing formation of the mature capsid [98, 176, 177].

### CAP-1

**Fig. 6:**
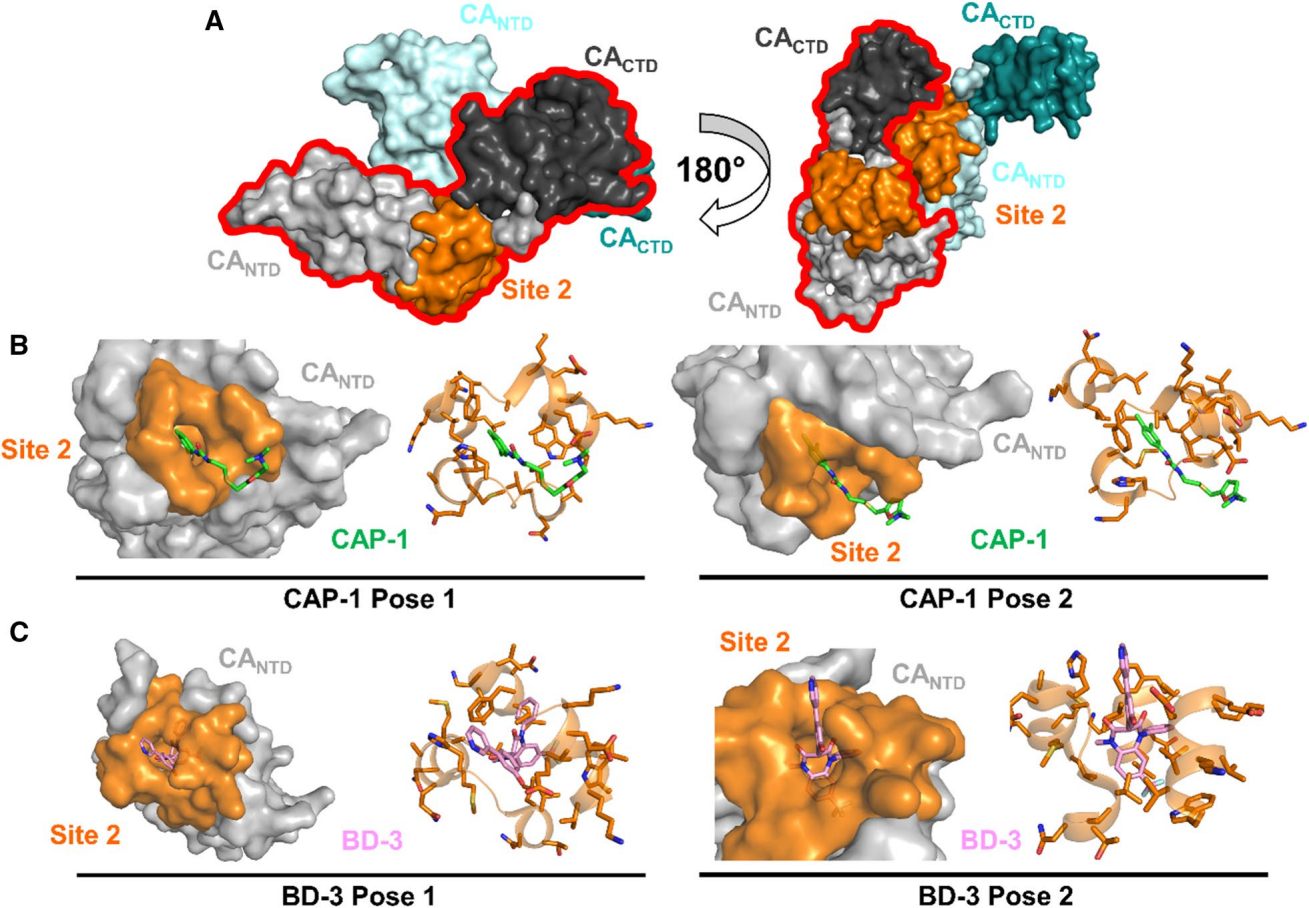
HIV-1 antivirals targeting the CAP-1 binding site. **A** Two CA monomers from a CA hexamer (PDB ID: 4XFX) with CA_NTD_s shown in light gray and light cyan, respective CA_CTD_s in dark gray and dark cyan, and binding site 2 in orange. One subunit is outlined in red. **B** NMR structures of CA_NTD_ in complex with CAP-1 (PDB ID: 2JPR; CAP-1 in green sticks). Left: surface and cartoon views of CA site 2 interacting with pose 1 of CAP-1. Right: surface and cartoon views of CA site 2 interacting with pose 2 of CAP-1. **C** Crystal structure of CA_NTD_ in complex with BD-3 (PDB ID: 4E91; BD-3 in pink sticks). Left: surface and cartoon views of CA site 2 interacting with pose 1 of BD-3. Right: surface and cartoon views of CA site 2 interacting with pose 1 of BD-3

CAP-1, *N*-(3-chloro-4-methylphenyl)-*N*0-(2-[((5-[(dimethylamino)-methyl]-2-furyl)-methyl)-sulfanyl]-ethyl) urea (Tables 1, 3 and Fig. 6), was the first discovered CA-targeting compound found in 2003 during a computational screen of small molecules against HIV-1 CA [178]. X-ray crystallography and NMR studies showed that CAP-1 binds to an induced hydrophobic pocket at the base of the CA_NTD_ helical bundle of the 5 α-helical apices of α1, α2, α3, α4, and α7 (Figs. 1, 6). The pocket is normally occupied by the aromatic side chain of F32 in structures of uninhibited CA hexamers, pentamers, and monomers. CAP-1 binding to this site induces conformational changes, displacing residues F32, H62, and Y145, and may disrupt the CA_NTD_/CA_CTD_ interface, leading to inhibition of mature capsid formation [98, 176]. Structural data supports previous findings that CAP-1 interfered with the ability of CA to form tubular assemblies, reduced HIV-1 infectivity by 95% at 100 μM, caused pleiomorphic particle formation that did not contain capsid, and did not affect viral entry, reverse transcription, integration, and GagPol cleavage [178]. Collectively, these results suggested that CAP-1 inhibited mature capsid formation, a novel antiviral mechanism at the time. Because of its low binding affinity to CA, CAP-1 was not developed any further. Nonetheless, these studies established the proof of principle for the potential utility of this binding site [107, 178] that may eventually lead to the development of more potent compounds that act by an analogous mechanism of action.

**Table 3:** Compounds targeting binding site 2: CAP-1 binding site

Name	Key interactions	Effects on stability and/or assembly	HIV-1 stage affected	IC_50_/EC_50_	CC_50_
CAP-1 [178]	N29, F32, S33, V36, M39, N53, V59, G60, H62, R143, M144, Y145	Decrease multimerization	Late	N/A	N/A
BD3 [177]	Helix α1	N/A	Late/early	0.4 µM	N/A
BM4 [177]	F32, H62 R162, D163, D166	N/A	Late/early	46 µM	N/A

*N/A* not available

Compound stabilizing effects on HIV-1 CA were determined by thermal shift assays and compounds effects on capsid assembly were determined by in vitro capsid assembly assays

### Benzodiazepines and benzimidazoles

Two other compound classes have been found to bind to the CAP-1 binding site located at the CA_NTD_. Benzodiazepines (BDs) (Tables 1, 3), specifically with a 1,5-dihydrobenzo[*b*][1,4]diazepine-2,4-dione scaffold, were identified during a capsid assembly screen for compounds that bind to CA_NTD_ (Site 2, Table 1) [179]. Subsequently, using a novel high-throughput screen assay based on the in vitro association of CA-NC subunits on immobilized oligonucleotides, other BDs and benzimidazoles (BMs) were shown to bind to this location (Site 2) [177]. Optimization of these compounds produced a series of BDs and BMs that had high antiviral potency against wild-type HIV-1 as well as HIV-1 strains resistant to RT, PR, and IN inhibitors. The top two compounds, BD3 and BM4, had EC_50_ values of 0.4 µM and 46 µM, respectively (Tables 1, 3). NMR and X-ray crystallographic studies showed that these two classes of small molecules bind to the CAP-1 binding site (Fig. 6). Interestingly, BDs and BMs acted with different modes of action. Using EM and virus release studies, BD molecules were shown to prevent virus release, whereas BMs inhibited capsid assembly. BD compounds were shown to bind further into the helical bundle, leading to a displacement of the α1 helix. The α1 helix contributes to inter-subunit interactions within the CA_NTD_ of hexamers and pentamers (Fig. 1) [98, 102]. Its displacement upon BD binding can explain the effects on virus replication and capsid morphology. In contrast to BDs, BMs do not bind as deeply into the pocket and seem to protrude outside of the site. This extended binding leads to clashes with residues R162, D163, and D166 of the neighboring hexamer’s CTD. Within the pocket, the specific BM displaces residues F32 and H62, similar to CAP-1, and shifts the loop between α3 and α4 (Fig. 1) [177]. All of these interactions resulted in a loss of mature capsid assembly. These findings reveal that BDs and BMs bind at the same pocket, but in distinct modes, resulting in different mechanisms of action.

Passage of HIV-1 with these compounds led to resistance mutations in the highly conserved binding pocket or at the CA_CTD_ [177]. This study demonstrated distinct patterns of selected resistance mutations with potent vs. weaker inhibitors and with BD vs. BM antivirals. Whereas the V36T and G61E mutants were selected using BD compounds, K30R and S33G were obtained only upon selection with BM antivirals. V27A/I changes were observed for the BD series only. Common resistance mutations outside of the pocket included T58I and G208I. Although these mutations either stabilized the mature capsid or decreased BD and BM binding affinity, many mutations also resulted in a less fit virus with impaired replication abilities or low resistance to the compounds [177]. Optimization of targeted antivirals for this pocket is challenging due to the pocket’s hydrophobicity and flexibility. Moreover, these compounds are not suitable antiviral agents due to their low solubility and limited bioavailability. Regardless, these studies identified two compound classes, BDs and BMs, that target the CA_NTD_ and reaffirmed that the HIV-1 capsid can be a target for viral inhibition.

## Site 3: The “twofold” binding site

CA is a critical component of viral maturation by driving Gag multimerization through dimeric interactions between CA_CTD_s from neighboring hexamers at the twofold symmetry interface [115] (Fig. 2B). The dimer interface involves the residues 179–192 and those of the 3_10_ helix (150–152) (Fig. 1) [99, 180, 181]. Additionally, the CA_CTD_ also contains the highly conserved MHR (residues 153–172), which is conserved among orthoretroviruses and participates in virus assembly. The MHR is not a part of the dimerization interface but maps to the binding region of some compounds that bind at this site [61, 97, 107, 108]. Mutagenesis studies have shown that the dimerization interface is critical for viral particle formation [123], and thus the high level of conservation of this site and the reliance on the CA_CTD_ for dimerization makes this site a potential site for drug development.

Several compounds that target this site are synthetic peptides. Though small-molecules dominate the HIV-1 therapeutic armamentarium, protein-based therapeutics have been characterized for many clinical purposes [182, 183]. As such, these peptides inhibit virion assembly by perturbing CA_CTD_ dimerization and subsequently HIV-1 maturation.

### CAI

**Fig. 7:**
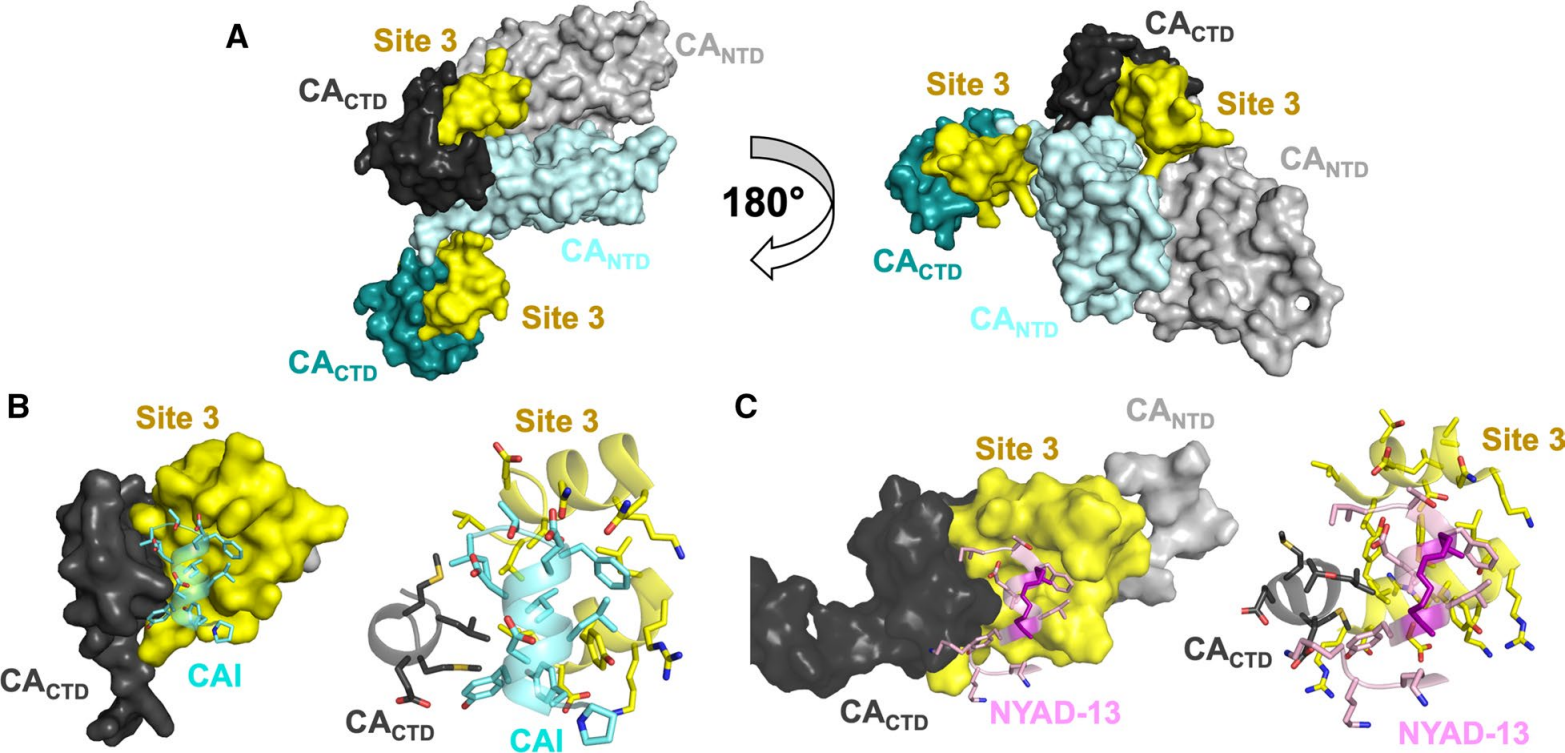
HIV-1 antivirals targeting the twofold binding site. **A** Two CA monomers from a CA hexamer (PDB ID: 4XFX) with CA_NTD_s shown in light gray and light cyan, with the respective CA_CTD_s in dark gray and dark cyan, and binding site 3 in yellow. **B** Crystal structure of CA_CTD_ in complex with CAI (PDB ID: 2BUO; CAI in cyan cartoon and sticks). Surface (left) and cartoon (right) views of CA site 3 interacting with CAI. **C** NMR structure of CA_CTD_ in complex with NYAD-13 (PDB ID: 2L6E; NYAD-13 in pink cartoon and sticks). Left: Surface (left) and cartoon (right) views of CA site 3 interacting with NYAD-13, which is stabilized through hydrocarbon stapling with (S)-2-(2ʹ-pentenyl) alanine (magenta cartoon and sticks)

One example of a peptide-based inhibitor is Capsid assembly inhibitor (CAI) that was discovered through a phage-display screen that identified 12-mer peptides with affinity for CA (Tables 1, 4) [184]. CAI (primary sequence: ITFEDLLDYYGP) was found to interact with the hydrophobic groove of the CA_CTD_ at the twofold interhexamer symmetry axis through an induced fit mechanism, resulting in an α-helical structure (Fig. 7) [100, 184]. Subsequent analysis revealed that CAI inhibits assembly of both immature and mature HIV-1 in vitro, however, it did not inhibit viral assembly in vivo [184]. Superposition of the CA_CTD_-CAI complex onto the CA_NTD_/CA_CTD_ interface of assembled CA suggested that CAI binding would likely sterically hinder the CA_NTD_/CA_CTD_ interaction. It was also suggested that CAI may also allosterically alter the CA_CTD_ dimer geometry, thus affecting propagation of the mature CA lattice [177].

**Table 4:** Compounds targeting binding site 3: twofold binding site

Name	Main Interactions	Effects on stability and/or assembly	HIV-1 Stage affected	IC_50_^a^/EC_50_	CC_50_
CAI [184]	Helix α1: 161–174 Helix α2: 180–192	Decrease multimerization	Early/late	N/A	N/A
NYAD-1 [185]	Helix α1: 161–174 Helix α2: 180–192	Decrease multimerization	N/A	4.0–15 µM^a^	57–80 µM
NYAD-13 [185]	I153, R154, Y164, A174, A177, V181, K182, A184, A194, I201, A204, G206, A209, E213, A217, Q219, V221, A228, V230	N/A	N/A	10.5 µM^a^	40.5 µM
CAC1 [188]	Helix α2^b^	N/A	N/A	N/A	N/A
NYAD-201 [190]	178–192^b^	Decrease multimerization	Early/late	2.8–5.2 µM^a^	> 115 µM
MKN-1A [191]	W184, M185^b^	N/A	N/A	8.0 µM	38 µM
16 [192]	N/A	N/A	N/A	5.0 µM^a^	N/A
Ebselen [193]	Helix α10: C198 Helix α11: C218	Decrease multimerization	Early	4.3 µM	37.9 µM
I-XW-053 [194]	R173, Ile37^b^	N/A	Early	48.4–93.6 µM^a^	> 100 µM

Compound stabilizing effects on HIV-1 CA were determined by thermal shift assays and compounds effects on capsid assembly were determined by in vitro capsid assembly assays

*N/A* not available

^a^Refers to IC_50_

^b^Refers to key interactions found through non-structural studies such as molecular docking

The early success of this compound led to the design of several improved derivatives. One such example is NYAD-1 (ITFxDLLxYYGP; x = (S)-2-(2ʹ-pentenyl)Ala), which strategically modified the sequence of CAI (Tables 1, 4) [185]. NYAD-1 replaces CAI residues D4 and G8 with a non-canonical amino acid, (S)-2-(2ʹ-pentenyl) alanine, to form a hydrocarbon staple. Hydrocarbon stapling is an established technique used to stabilize the secondary structure of helical peptides by crosslinking the i and i + 4 positions with an olefinic chain via alanine analogues [186]. NYAD-1 formed a stable helix in solution and was cell-permeable, overcoming a large issue of CAI [184, 185]. Further, NYAD-1 binds the CA_CTD_ from residues 169–191, the same interface as CAI, and specifically disrupted the formation of mature HIV-1 particles in a dose-dependent manner with an EC_50_ ~ 4–15 μM and a CC_50_ > 135–300 μM [185]. To further study the stabilized helical structures, NYAD-13 was derived from NYAD-1 by replacing the C-terminal proline with three lysine residues (ITFxDLLxYYGKKK; x = (S)-2-(2ʹ-pentenyl)Ala) (Tables 1, 4) [185, 187]. The positive charges increased the solubility of the peptide and facilitated structural studies. The high-resolution NMR structure of the CA_CTD_/NYAD-13 complex showed that the intermolecular interactions were mediated by the packing of hydrophobic side chains at the buried interface and remained unperturbed due to the hydrocarbon stapling chain. NYAD-13 has antiviral activity (IC_50_ ~ 10.5 μM, CC_50_ = 40.5 μM) [185, 186]. These structural analyses provided further insight for the design of higher affinity selective inhibitors of HIV-1 particle assembly.

### CAC1

The antiviral peptide CAC1 (primary sequence: EQASQEVKNWMTETLLVQNA) was designed based on the premise that helical mimics of CA would inhibit capsid assembly (Tables 1, 4) [188]. Its sequence mimics helix α9 of the twofold symmetry interface. CAC1 was shown to bind to CA_CTD_ using NMR, size exclusion chromatography, thermal denaturation measurements, and circular dichroism (K_d_ of 50 μM). CAC1 binding is thought to disrupt the dimer interface, resulting in the loss of key interactions that are crucial for capsid assembly. Bocanegra et al*.* subsequently designed other helical mimics, CAC1C, CAC1M, and H8, aiming to increase solubility and binding affinity. Using 2D ^1^H–^15^N HSQC-NMR, CAC1C (ESASSSVKAWMTETLLVQNA) and CAC1M (SESAASSVKAWMTETLLVANTSS) were shown to bind the CA_CTD_ at the same site as CAC1 [189]. The binding affinities of CAC1C and CAC1M were higher than that of CAC1, with K_d_s of 19 μM and 8 μM, respectively. H8 (KEPFRDYVDRFYKTLRAEQ) was designed to mimic helix α8 of the CA_CTD_ and was able to inhibit viral assembly in vitro, albeit to a lesser extent than CAC and CACM. Inhibition of HIV-1 by CAC1/CAC1M, H8, and CAI (mentioned above), alone and in combination, was then tested in vitro and in vivo and these compounds worked best in combination with some synergistic relationships identified [188]. While the twofold binding site is potentially important for viral replication, the available peptides do not have potent antiviral activity.

To further stabilize the helical mimics, NYAD-201 (AQEVKxWMTxTLLVA; x = (S)-2-(2ʹ-pentenyl)Ala) was designed in a similar fashion to CAC1 by modifying the CA_CTD_ dimerization domain (SQEVKNWMTETLLVQ) (Tables 1, 4) [190]. N6 and G11 from the interhexameric dimer interface sequence were replaced to allow hydrocarbon stapling to generate a more stable, α-helical, cell permeable antiviral with an EC_50_ of 2.8–5.2 μM and a CC_50_ > 115 μM [190]. The observation that relatively weak CA binders, such as NYAD-201, are sufficient to dissociate and deform the virion cores offers encouragement for the exploration of a broader class of peptide antivirals for targeting CA.

### Other antivirals

#### MKN-1A

A highly conserved Tryptophan (W)–Methionine (M) dipeptide (W184/M185) motif in H9 of CA_CTD_ is engaged in hydrophobic interactions with the corresponding W184/M185 residues of a CA in a neighboring hexamer, at the twofold symmetry axis. Not surprisingly, mutations of these residues impair viral assembly [123, 191]. A small molecule was selected by in silico screening as a peptidomimetic compound of W184/M185 in the interaction site. It was synthesized and its anti-HIV-1 activity was demonstrated. The lead compound from this study was MKN-1A (EC_50_ = 8 µM; CC_50_ = 38 µM) (Tables 1, 4). Of note, the chirality of this molecule was important, as an MKN-1A diastereomer had decreased potency. Additionally, the trypsin-based indoyl and the methionine-based sulfidyl groups were essential for antiviral activity [191]. Hence, these efforts further confirmed that this site is a potential therapeutic target for subsequent drug design.

#### Compound 16

Machara et al*.* were the first to identify a set of small molecules that bind the CAI pocket [192]. Their high-throughput screening method was based on the amplified luminescence proximity assay system (Alphascreen). A high signal was produced when CAI attached to a donor bead came into close proximity of CA_CTD_ on an acceptor bead. This signal could then be competed by molecules that were able to interact with CA_CTD_. Out of > 7000 compounds screened, they identified a family of 2-arylquinazolines able to compete with CAI and presumably bind at the same binding site. Compound 16 was shown to be the most potent 2-arylquinazoline (IC_50_ = 5 µM) (Tables 1, 4) and was also shown to inhibit HIV-1 in tissue culture at low micromolar concentrations.

#### Ebselen

Ebselen was identified from a high-throughput screen for inhibitors of CA_CTD_–CA_CTD_ dimerization (Tables 1, 4) [193]. Liquid chromatography–electrospray ionization–mass spectrometry (LC–ESI–MS) confirmed that ebselen binds covalently to CA by forming a selenylsulfide (-Se-S-) bond with cysteines C198 and C218 at the CA_CTD_, thus promoting a conformation of an ebselen-linked monomer that is susceptible to aggregation. While ebselen may not directly interact with residues at the twofold interface, NMR studies confirmed that its binding changes the chemical shift of residues in helix α9, which is involved in CA dimerization. Ebselen binds with an IC_50_ of 47 nM and has antiviral activity with an EC_50_ of 4.3 μM [193].

#### I-XW-053

Kortagere et al*.* [194] used a hybrid structure-based screening method and identified 900 compounds of interest. Further docking and scoring analysis and a single-round infection study identified CK026, which was modified to yield DMJ-I-073, I-XW-091, and I-XW-053 (Tables 1, 4). The most potent of the series, I-XW-053, appear to have modest potency (48.4 to 93.6 µM), (Tables 1, 4). Mutational analysis identified residues R173 and I37 to be essential for I-XW-053 binding [194] suggesting proximity to α9 helix, which is at the twofold interface.

## Site 4: Apical binding site

### Benzimidazoles (BM)

**Fig. 8:**
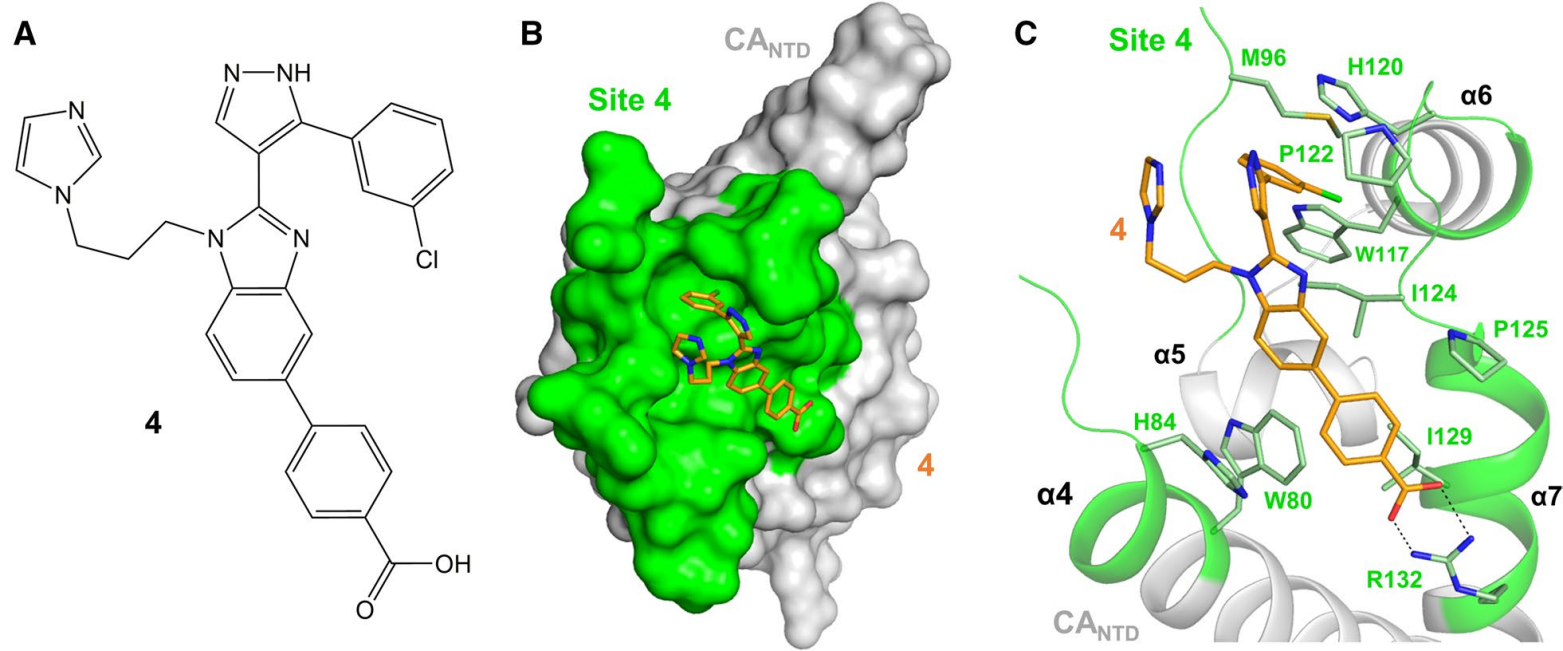
HIV-1 antivirals targeting the apical binding site. **A** Chemical structure of benzimidazole compound 4 (from [195]). **B** Crystal structure of CA_NTD_ in complex with 4 (PDB ID: 4E91; compound 4 in orange sticks) in surface view. CA_NTD_ is shown in light gray, with site 4 colored in green. **C** Close-up view of compound 4 at site 4, colors as in (**B**). Residues having strong hydrophobic interactions with compound 4 are shown as light green sticks. Hydrogen bonds with the side chain of R132 are shown as black dotted lines

As mentioned above, some BM compounds can interact with CA at the binding site 2 (Fig. 8, Tables 1, 5). However, other BMs can bind to a unique capsid pocket located at the apical binding site of CA_NTD_, near the CypA-BL, in this review referred to as binding site 4 [195]. The CA residues that comprise this relatively shallow binding pocket and interact with these compounds were revealed using ^1^H–^15^N HSQC-NMR and confirmed using X-ray crystallography. They are at the top of helix α4, the base of the CypA-BL, within helices α5 and α6, the loops joining helices α5, α6, and α7, and at the top of helix α7 (Figs. 1, 8). While the BM compounds bind near the CypA-BL, it was reported that CypA does not compete with binding of BM inhibitors to the CA_NTD_. The EC_50_ and CC_50_ values of these small molecules ranged from 0.95–2.8 μM and 57–80 μM, respectively. Interestingly, molecules of this type were also able to bind CA in the presence of CAP-1. However, a significant proportion of the apical binding site is made from residues that are polymorphic among various strains, H120 for example in Fig. 8C. These polymorphisms were shown to affect the potency of these inhibitors, eliminating them as candidates for possible HIV-1 therapies. Nonetheless, these studies established the apical binding site as a novel HIV-1 CA binding site and further support that CA can be simultaneously targeted by more than one CA-targeted antiviral (BM and CAP-1) [195].

**Table 5:** Compounds targeting binding site 4: apical binding site

Name	Key interactions	Effects on stability and/or assembly	HIV-1 stage affected	IC_50_^a^/EC_50_	CC_50_
Compound 1 [195]	I2, E53, L83, I104, A105, T107, T108, T110, Q114, G116, W117, T119, H120, N121, V126, G127, R132	N/A	N/A	6.1 µM^a^	N/A
Compound 2 [195]	N/A	N/A	N/A	1.2 µM^a^	N/A
Compound 3 [195]	N/A	N/A	N/A	1.4 µM^a^	N/A
Compound 4 [195]	W80, M96, G98, W117, H120, P122, P123, I124, P125, I129, R132	N/A	N/A	1.2 µM^a^	N/A
Compound 5 [195]	N/A	N/A	N/A	2.8 µM	> 80 µM
Compound 6 [195]	N/A	N/A	N/A	0.95 µM	57 µM

*N/A* not available

^a^Refers to IC_50_

## Site 5: Central pore binding site

**Fig. 9:**
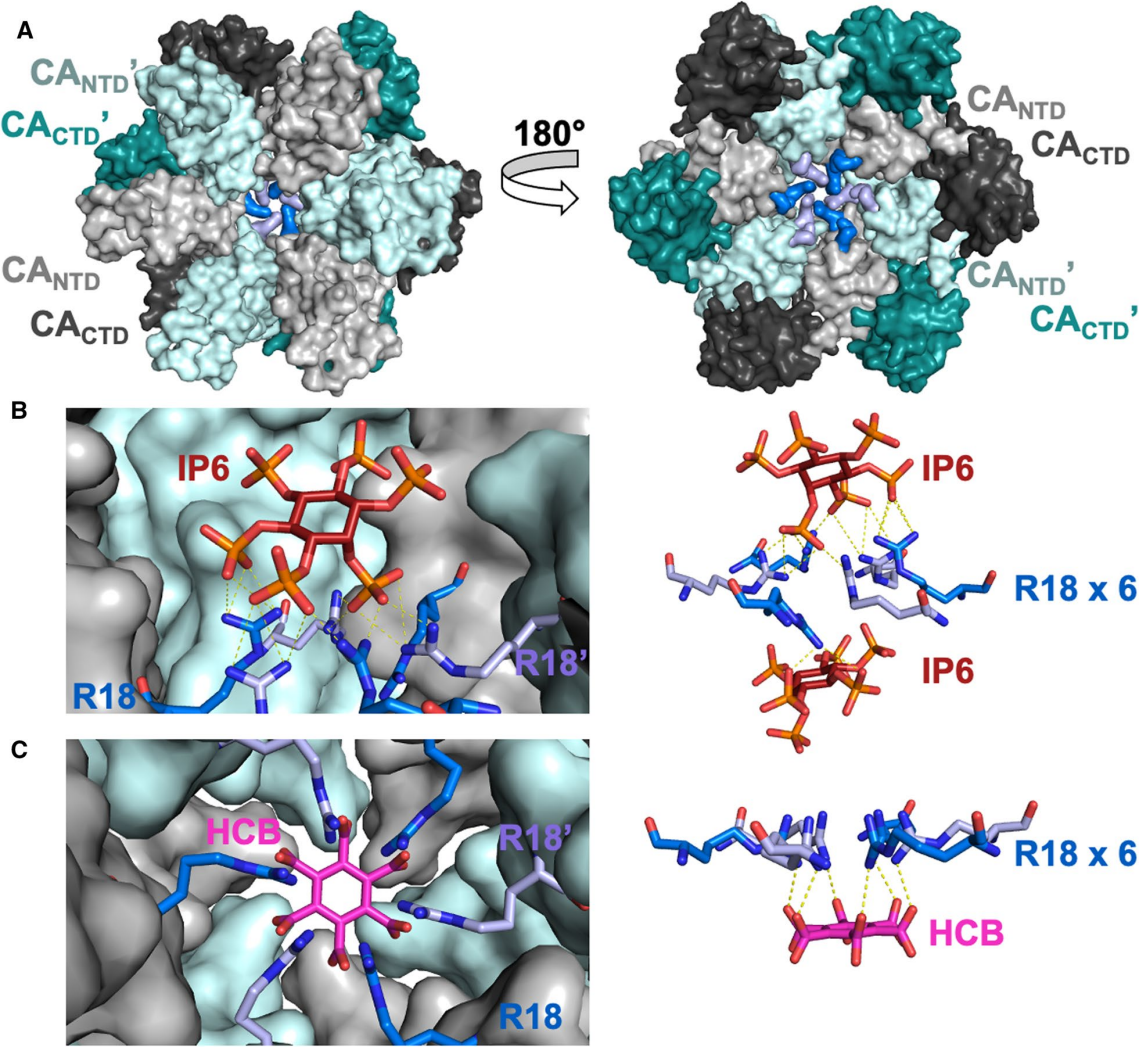
HIV-1 CA central pore binding site. **A** CA hexamer (PDB ID: 4XFX) is shown with CA_NTD_s in light gray and light cyan, CA_CTD_s in dark gray and dark cyan, and R18 side chains at the sixfold symmetry axis in alternating blue and light blue colors. **B** IP6 (brown sticks) is shown bound to CA hexamer (PDB ID: 6BHT). Right, IP6 above and below R18 ring. **C** Hexacarboxybenzene (HCB; magenta sticks) bound to CA hexamer (PDB ID: 5HGP). Right, HCB interacting with R18

At the center of the sixfold symmetry axis in CA hexamers, there is a positively charged central pore lined by six arginine residues (R18) from each of the CA_NTD_s in the CA hexamer, having the appearance of an iris [196] (Fig. 9). At the top of the pore, the conformation of the flexible N-terminal β-hairpin changes in a pH-dependent manner, with the pore appearing open under acidic conditions and blocked by the hairpin under basic conditions. The pore has been proposed to serve as a gate through which dNTPs enter inside the core [112, 196]. Abolishing positive charges impaired viral infectivity and reverse transcription in a dose-dependent manner.

IP6, the anionic metabolite essential for immature Gag assembly, also interacts with the ring of the six R18 residues in the mature capsid (Table 1) [80]. Additional interactions were recently reported for a more internal, basic ring made by six K25 residues, which based on its observed binding of inositol pentaphosphate (IP5) was speculated to also bind IP6 [109]. Crystallographic studies suggest that IP6 assumes conformations that are almost parallel or at an angle, with respect to the ring of six R18 or K25 residues. Of note, based on molecular dynamics (MD) simulations it was proposed that IP6 assumes a binding mode perpendicular to the R18 ring, thus allowing unhindered dNTP entry through the pore (Fig. 9B) [80, 112]. While the central pore binding site has been proposed to be the binding site in the mature capsid, simulation studies report that IP6 may additionally bind to other pockets, including site 1 [197].

### Carboxybenzenes

It was reported that the symmetrical, polyanionic carboxybenzene compounds bind to the R18 ring within the six-fold axis [196] (Fig. 9). The potency of these compounds was low in in vitro studies, as the compounds decreased, but did not abolish, reverse transcription. The most effective of these molecules is hexacarboxybenzene (HCB), also known as benzenehexacarboxylic acid or mellitic acid (Tables 1, 6). MD simulations found that HCB has a preference to lay parallel to the R18 ring, in contrast to IP6 that seems to generally bind at an angle (Fig. 9C) [112]. The parallel positioning of HCB was proposed to block the pore and prevent dNTPs from entering the interior of capsid where reverse transcription takes place [112, 196]. Although these compounds are not useful as potential therapeutics, they provide support to the hypothesis that the central pore binding site may be useful for future drug discovery efforts.

**Table 6:** Compounds targeting binding site 5: central pore binding site

Name	Key interactions	Effects on stability and/or assembly	HIV-1 stage affected	IC_50_/EC_50_	CC_50_
IP6 [80]	R18, K25	Stabilize	Early/late	N/A—Not an antiviral	N/A
HCB [196]	R18	N/A	N/A	N/A	N/A
ACAi-028 [198]	Q13, T19, S16^b^	Destabilize	Early	0.55 µM	N/A

Compound stabilizing effects on HIV-1 CA were determined by thermal shift assays and compounds effects on capsid assembly were determined by in vitro capsid assembly assays

*N/A* not available

^b^Refers to key interactions found through non-structural studies such as molecular docking

### ACAi-028

Recently a small molecule, ACAi-028, was shown to inhibit HIV-1 by binding to a hydrophobic pocket located at the CA_NTD_, interacting with the β-hairpin end, the flexible linker, and the front edge of helix α1 [198] (Tables 1, 6, Fig. 1). Amano et al*.* found that the addition of a small amino acid sequence in the CA_NTD_, specifically at R18 and T19, resulted in CA degradation [199]. Based on this information, Chia et al*.* hypothesized that small molecules binding at this area would affect the properties of the core [198]. Using in silico docking, they identified 40 compounds that are predicted to bind this hydrophobic pocket and inhibit HIV-1 replication with relatively low cytotoxicity. ACAi-028 emerged as the top candidate (EC_50_ = 0.55 µM). This small molecule was proposed to form hydrogen bonds with Q13, T19, and the backbone of S16. These results suggest that ACAi-028 may bind to monomeric HIV-1 CA. ACAi-028 was shown to inhibit the early stages of HIV-1 replication, likely the capsid uncoating process. ACAi-028 is unable to inhibit HIV-2 likely because of differences in amino acid sequence (residues 2–15) at the proposed binding sites and a shallower hydrophobic pocket. This study highlights the HIV-1 CA_NTD_ hydrophobic pocket and β-hairpin as potential antiviral targets and presents small molecules that may target this site [198].

## Conclusion

Despite the great success of ART, complications can emerge during this life-long treatment. Antiretrovirals put a selective pressure on HIV-1 to develop mutations that cause drug resistance. To combat the emergence of drug resistance, there is continued interest in developing novel therapeutic targets. Of the possible HIV-1 proteins, CA is an excellent candidate for antiviral design because of its influential, and often indispensable roles in HIV-1 cytoplasmic trafficking, immune evasion, reverse transcription, nuclear entry, integration, assembly, and maturation.

As such, in 2003 CAP-1 was the first compound reported to target CA and bind in what we described here as Site 2. Although CAP-1 did not have clinical success due to its low binding affinity, this molecule experimentally demonstrated that CA is a valid target for drug design. Almost 20 years later, the field of HIV-1 capsid inhibitors has expanded greatly, as we have discussed over 40 small molecules and peptides that exert antiviral activity by targeting five functional binding sites on HIV-1 capsid.

Of the five CA antiviral binding sites, site 1 has been targeted with marked success by compounds such as PF74 and GS-6207. In particular, the development of PF74 by Pfizer was a major landmark in the development of CA-targeting compounds. While it never reached the clinic, PF74 demonstrated sub-micromolar potency and serves as the backbone for some of the most promising current compounds, including PF74 analogues with increased potency, particularly against CA mutants that display resistance to PF74. The core peptidic backbone of PF74 is also at the heart of the most promising CA targeting compound to have been developed thus far, GS-6207, developed by Gilead Sciences. GS-6207 is extremely potent and is currently in phase 2/3 clinical trials. Although this compound is the most successful capsid inhibitor to-date, drug resistant mutants have been selected in passaging experiments and it remains to be seen whether resistance will emerge during therapeutic use. Further research into compound binding at Site 1, and the impact of resistance associated mutations, will promote the development of antivirals with improved resistance profiles via structure-based design. Overall, HIV-1 capsid inhibitors are a diverse set of compounds with excellent therapeutic potential. Each of the five targeted sites described here contributes to at least one of the many functions of CA in HIV-1 replication. Mutations at these sites often impair viral fitness, demonstrating their usefulness as antiviral targets. Continued work on capsid inhibitors and their targeted sites will further our understanding of HIV-1 biology and aid in the development of next generation antiretroviral drugs.

## Future perspectives

The new developments in capsid-targeting antivirals have generated excitement and have validated HIV-1 CA monomers and the HIV-1 capsid core as a therapeutic target. Future work may focus on the development of later generation antivirals to address challenges of solubility, metabolic stability, and barrier to resistance, while maintaining potency. Importantly, in a recent development, Gilead Sciences and Merck & Co. announced investigational treatment combinations of lenacapavir (GS-6207) and islatravir (EFdA), enabling powerful combinations of long-acting therapeutics that target HIV-1 CA and reverse transcriptase [200–203].

## Data Availability

Data sharing is not applicable to this article as no datasets were generated or analyzed during the current study.

## Abbreviations

AIDS: Acquired immune deficiency syndrome
ART: Antiretroviral therapy
BD: Benzodiazepines
BM: Benzimidazoles
CA: Capsid protein or domain
CA_CTD_: Capsid protein C-terminal domains
CAI: Capsid assembly inhibitor
CA_NTD_: Capsid protein N-terminal domains
CPSF6: Cleavage and polyadenylation specificity factor subunit 6
Cryo-EM: Cryogenic-electron microscopy
Cryo-ET: Cryogenic-electron tomography
CypA: Cyclophilin A
CypA-BL: Cyclophilin A-binding loop
dNTPs: Deoxynucleotide triphosphates
EFdA: Islatravir
Env: Envelope protein
FG: Phenylalanine-glycine dipeptide
GS-6207: Lenacapavir
HCB: Hexacarboxybenzene
HIV: Human immunodeficiency virus
HSQC: Heteronuclear single quantum coherence
IN: Integrase
INSTI: Integrase strand transfer inhibitors
IP5: Inositol pentaphosphate
IP6: Inositol hexaphosphate
LC–ESI–MS: Liquid chromatography–electrospray ionization–mass spectrometry
MA: Matrix protein or domain
MD: Molecular dynamics
MDR: Multiple drug resistance
MHR: Major homology region
NC: Nucleocapsid protein or domain
NMR: Nuclear magnetic resonance
NNRTI: Non-nucleoside reverse transcriptase inhibitors
NPC: Nuclear pore complex
NRTI: Nucleoside reverse transcriptase inhibitors
PF74: PF-3450074
PI: Protease inhibitors
PI(4,5)P_2_: Phosphatidyl-4,5-bisphosphate
PR: Protease protein or domain
RNP: Ribonucleoprotein
RT: Reverse transcriptase
SP: Spacer peptide
TAF: Tenofovir alafenamide
α: α-Helix
βPin: β-Hairpin
